# The gut microbiome: neurological development and disease

**DOI:** 10.1007/s00415-022-11334-1

**Published:** 2022-08-17

**Authors:** Latif Miah, Neil Robertson

**Affiliations:** 1 Department of Neurology, University Hospital Wales, Cardiff, CF14 4XN UK; 2 Institute of Psychological Medicine and Clinical Neuroscience, Cardiff University, Cardiff, UK

## Introduction

The concept of an association between gut microbiota and brain function has been postulated for many years. However, to date any association remains poorly defined and research in this field is at a relatively early stage. In particular, the production of consistent data for analysis together with methodology to account for confounding variables which affects the microbiome composition, such as diet and exercise, remain barriers to overcome. However, a new focus has developed in this area, particularly in animal models where these factors can be more effectively controlled. More recently microbiota dysbiosis has been implicated in a range of neurological diseases including Alzheimer’s disease, with some evidence that dietary induced changes in microbiome composition alter dietary metabolites, which in turn may influence neuronal function. All of which offers the possibility of a novel therapeutic approach for these disorders.

The first paper discussed this month implicates altered gut microbiomes in early life with aspects of neurogenesis and synaptic transmission together with autism spectrum disorder (ASD)-like behaviour and hippocampal dysfunction in murine models. The second paper further extends this theme by evaluating gut microbiota in schizophrenia as a biomarker of disease. The final paper explores genetic aspects of the gut-brain axis revealing that specific gut sensory neurons may differentially control feeding and glucose metabolism, with subsequent links to obesity and obesity-related neurological conditions such as idiopathic intracranial hypertension.

## Gut dysbiosis impairs hippocampal plasticity and behaviors by remodeling serum metabolome

Metabolites derived from the gut microbiome, such as short chain fatty acids, serotonin and tryptophan, are thought to be key players in the signalling between the Central Nervous System (CNS) and the gut. Furthermore, the infant microbiome is significantly correlated with fine motor and social skills. Previous animal studies have suggested that absence of a microbiome adversely affects the integrity of the blood–brain barrier and leads to physiological changes to the prefrontal cortex, hippocampus and amygdala.

Liu et al. established a gut dysbiosis mouse model using selected antibiotics for four weeks. Two weeks after the final dose, faecal matter underwent 16S rDNA high-throughput sequencing to determine microbiome composition. The mice were the subjected to a series of behavioural tests focussed on spatial learning/memory deficits and behavioural impairments including anxiety. Specifically, Firmicutes were reduced whilst proteobacteria increased. Oher bacteria, which negatively correlated with cognition, were also found to be elevated. Long Term Potentiation (LTP), the process underpinning learning and memory, was found to be impaired in antibiotic treated mice as compared to controls, indicating a reduction in synaptic plasticity. Further reinforcing this was an observed change on dendritic spine morphology in the antibiotic-treated mice. In addition, by labelling hippocampal progenitor cells with relevant antibodies, gut dysbiosis was shown to impair adult neurogenesis as compared to control.

Hippocampal transcriptomic analysis also revealed impaired expression of Immediate Early Genes (IEGs) in the dentate gyrus of antibiotic-treated mice associated with adverse effects in plasticity, learning and memory. Gene expression of LCN2 and LRG1 associated with impaired plasticity and memory were significantly increased. The researchers also found that faecal transplantation to restore the microbiome of the treatment group resulted in significant improvement of behavioural deficits, neurogenesis and LTP and that expression of IEGs appeared restored.

Investigating another bacterial metabolite, naïve mice were treated with 4-methylphenol for 2 weeks. Exposure to this metabolite downregulated IEGs whilst increasing expression of LCN2 and LRG1, inhibiting spine maturation of hippocampal neurons. In addition, upregulated inflammatory/apoptotic signals led to neuronal death in the dentate gyrus and CA1 region of the hippocampus.

*Comment*: The authors show that there is a critical neurodevelopmental window shortly after birth, where dysbiosis can have severe long-term consequences to hippocampal function and so learning and memory via altered microbiomes and their metabolites. This suggests a potential avenue of therapeutic intervention for neurodevelopmental disorders.

Liu G et al. Gut Microbes. 2022 Jan–Dec;14(1):2104089. https://doi.org/10.1080/19490976.2022.2104089. PMID: 35876011; PMCID: PMC9327780.

## Intestinal microbes in patients with schizophrenia undergoing short-term treatment: core species identification based on co-occurrence networks and regression analysis

Gut microbiota has been associated with cognitive impairment, and is bidirectionally linked to brain function via neural, endocrine and immune pathways. Having already induced schizophrenia in mouse models via faecal transplantation from human schizophrenic donors (noting that lower glutamate and higher glutamine & GABA were observed in the hippocampus and specific microbes were closely associated with schizophrenia such as actinobacteria), Xiang et al. aimed to determine if severity of disease could be correlated to gut dysbiosis, and whether specific bacteria can act as biomarkers. The investigators identified pathogen-free mice and performed faecal transplantation from validated human inpatient schizophrenic donors both before and 14–19 days after treatment of schizophrenia with antipsychotics.

DNA extraction and Gene sequencing analysis of the faecal matter identified statistically significant differences in microbiome diversity in the 2 groups: before treatment (BT) and after treatment (AT). Through random forest algorithms and co-occurrence network and netshift analysis, key players were identified. AT levels of Faecalibacterium, Butyricicoccus, Oscillospira, Prevotella, and Roseburia increased whilst Rothia decreased. Faecalibacterium is a common bacterial signature in disorders such as depression and schizophrenia. Other bacteria with increased expression on gene sequencing have also been implicated in behavioural and psychological dysfunction with lower levels indicating greater disease severity, whilst Rothia has been associated with inflammatory response.

A battery of behavioural tests were then conducted on the BT and AT mice which demonstrated improved memory, sociability and interaction in the AT group as compared to BT mice. Again, lending strength to the assertion that the microbiome is closely linked to neurodevelopment and disease.

*Comment*: This study has revealed gut microbiome changes in response to antipsychotic treatment in male Schizophrenic inpatients of Han Chinese nationality. It has further demonstrated that composition of the BT and AT microbiome differ and has identified potential biomarkers of disease severity. This offers some evidence for an alternative therapeutic approach for these disorders and lends further credence to a role for the gut-brain axis in neurodevelopment and disease.

Xiang M et al. Front Microbiol. 2022 Jun 17;13:909729. https://doi.org/10.3389/fmicb.2022.909729. PMID: 35783418; PMCID: PMC9247572.

## Gut–brain communication by distinct sensory neurons differently controls feeding and glucose metabolism

Communication in the gut-brain axis is facilitated by afferent sensory neurons though a mechanism of action that is not fully understood. In particular, neurons communicate with the brain to affect sensations of satiety as well as regulation of glucose metabolism and insulin resistance. This may have a downstream influence on conditions such as obesity and closely associated neurological conditions, including idiopathic intracranial hypertension (IIH). Borgmann et al. used intersectional genetic manipulation to explore these feeding and glucoregulatory roles.

To accurately isolate sensory neurons involved in glucoregulation and explore related neural networks, Borgmann et al. bred triple transgenic mice by crossing three sets of mouse lines; The Nav1.8-p2a-Dre line (Nav1.8 being a sodium channel expressed only in sensory neurons), the intersectional Ai66 line and multiple Cre-expressing lines. The resultant lineage expressed tdTomato only within the sensory neurons of interest expressing Cre and Dre recombinase. This resulted in a validated intersectional genetic targeting of molecularly defined sensory neurons facilitating study of related networks.

Within this defined vagal afferent population, glucagon-like peptide-1 receptor (GLP1R) and GPR65 expressing neurones were investigated for regulatory function. The authors demonstrated that GLP1R-expressing neurons generated anorexigenic signals to parabrachial nucleus neurons that control meal termination. Meanwhile, activating GLP1R-expressing neurones improves glucose tolerance whilst inhibition elevates blood glucose levels independent of food intake. Contrastingly, stimulation of GPR65-expressing neurones increases hepatic glucose production, activating parabrachial neurons that control normoglycemia. Thus, distinct gut-innervating sensory neuron populations differentially control feeding and glucoregulatory functions and may provide specific targets for metabolic control.

*Comment*: The authors identify a novel target for intervention in IIH, through targeted action on neural networks involved in glucoregulation and feeding/satiety in the gut. This would act to help regulate healthy eating in obese individuals, facilitating weight loss which remains the most effective treatment for IIH. However, it should be noted that the marker used to identify the sensory neurons Nav1-8, may be absent in selected sensory neuron populations. In addition, the sensory neurons targeted are not exclusive to the gut so that neuronal populations from other organs may also be relevant.

Borgmann D et al. Cell Metab. 2021 Jul 6;33(7):1466–1482.e7. https://doi.org/10.1016/j.cmet.2021.05.002. Epub 2021 May 26. PMID: 34043943; PMCID: PMC8280952.

